# N‐acetylcysteine improves diabetic associated erectile dysfunction in streptozotocin‐induced diabetic mice by inhibiting oxidative stress

**DOI:** 10.1111/jcmm.17394

**Published:** 2022-05-20

**Authors:** Zhen Ma, Wenzhen Wang, Chao Pan, Cuiqin Fan, Ye Li, Wenjing Wang, Tian Lan, Fangxin Gong, Changbo Zhao, Zichao Zhao, Shuyan Yu, Mingzhen Yuan

**Affiliations:** ^1^ 12589 Department of Urology Shandong Provincial Hospital Cheeloo College of Medicine Shandong University Jinan China; ^2^ 12589 Department of Urology The Second Hospital, Cheeloo College of Medicine Shandong University Jinan China; ^3^ 12589 Department of Physiology School of Basic Medical Sciences Shandong University Jinan China; ^4^ Department of Urology Liaocheng People's Hospital Shandong China

**Keywords:** diabetes mellitus, erectile dysfunction, N‐acetylcysteine, oxidative stress

## Abstract

Oxidative stress appears to play a role in the pathogenesis of diabetes mellitus erectile dysfunction (DMED). This study aimed to investigate the effect of N‐acetylcysteine (NAC) on DMED in streptozotocin‐induced diabetic mice and to explore potential mechanisms. In the present study, we show that an erectile dysfunction is present in the streptozotocin‐induced mouse model of diabetes as indicated by decreases in intracavernous pressure responses to electro‐stimulation as well as from results of the apomorphine test of erectile function. After treatment of NAC, the intracavernous pressure was increased. In these DMED mice, oxidative stress and inflammatory responses were significantly reduced within the cavernous microenvironment, while activity of antioxidant enzymes in this cavernous tissue was enhanced after NAC treatment. These changes protected mitochondrial stress damage and a significant decreased in apoptosis within the cavernous tissue of DMED mice. This appears to involve activation of the nuclear factor erythroid 2‐like‐2 (Nrf2) signalling pathway, as well as suppression of the mitogen‐activated protein kinase (MAPK) p38/ NF‐κB pathway within cavernous tissue. In conclusion, NAC can improve erectile function through inhibiting oxidative stress via activating Nrf2 pathways and reducing apoptosis in streptozotocin‐induced diabetic mice. NAC might provide a promising therapeutic strategy for individuals with DMED.

## 1 INTRODUCTION

Erectile dysfunction (ED), defined as the inability to attain or maintain a penile erection sufficient for successful vaginal intercourse, is one of the most common sexual dysfunctions.^1^ Diabetes mellitus (DM) is the second most common risk factor for ED, which is present in 50%–75% of diabetics,^2^ and the number of men with diabetes‐associated ED continues to grow rapidly. However, no effective avenues currently exist for the treatment of diabetes mellitus‐induced erectile dysfunction (DMED). The first‐line treatment for ED consists of Phosphodiesterase type 5 inhibitors (PDE5i), which are effective for most ED patients. However, the efficacy of this treatment is much lower in patients with DMED.^3^, ^4^ There have been several studies directed at examining potential therapies for DMED patients, including gene therapy, transplantation of stem cell, anti‐PDF5 therapy and low energy shock wave therapy.^5^ Unfortunately, none of these methods have proved fully effective in the treatment of DMED. Therefore, the pathogenesis of DMED may result from an array of more complicated mechanisms.^6^

Normal penile erection is considered a complicated biopsychosocial process, which incorporates several systems including psychological, endocrine, vascular and neurological.^7^ In addition, the involvement of NO release from the endothelium and parasympathetic input are considered as vital components in penile erection. NO induces smooth muscle relaxation via activation of soluble guanylyl cyclase and the accumulation of cyclic guanosine monophosphate (cGMP) in cavernosal smooth muscle cells.^8^ The complexity of DMED may reside in changes within its microenvironment that then affect all of the above listed key factors involved in erection.

It has been reported that increased oxidative activity and expression of inflammatory markers, such as endothelin‐1 (ET‐1) and intracellular adhesion molecule‐1 (ICAM‐1), are present in DMED patients.^9^, ^10^ Inflammation and oxidative stress due to chronic hyperglycaemia exert damaging effects upon the vasculature, nerves and NO, which then seriously impairs penile function. Such destructive effects involve a cascade of reactions, with the initial component being the formation of reactive oxygen species (ROS) resulting in increased NO scavenging.^11^ Subsequently, this accumulation of ROS affects several intracellular pathways associated with vascular endothelial function, such as decreased eNOS expression or activity, which impairs endothelium‐dependent vasorelaxation and thus ED.^12^ Finally, this chronic inflammation and oxidative stress simultaneously weakens neurological responses involving non‐adrenergic non‐cholinergic nerves (NANC), resulting in a decrease of nNOS activity and corporal relaxation dysfunction.^13^

The antioxidant, N‐acetylcysteine (NAC), has been reported to play an essential role in regulating the redox environment of cells.^14^ However, the impact of NAC on erectile function in DMED animals has not been studied. Based on these findings, we hypothesized that NAC might be a potential drug for the maintenance of erectile function in diabetic patient Therefore, the goal of the present study was to investigate potential changes in the inflammation‐oxidative stress microenvironment of penile cavernous tissue within a mouse model of DMED. Taken together, these findings suggest that antioxidants may serve as a potential therapeutic strategy in the treatment of DMED.

## 2 MATERIALS AND METHODS

### 2.1 Animals

Male C57BL/6J specific‐pathogen‐free mice (8 weeks of age) were obtained from the Qilu Medical College Laboratory Animal Center of Shandong University. Mice were housed in groups of four per cage under standard laboratory conditions with free access to food and water and maintained under a 12h light/dark cycle (lights on 6:30 a.m., lights off 6:30 p.m.) for at least one week prior to experimental procedures. The study was conducted according to the guidelines of the Declaration of Helsinki and approved by the Animal Care and Use Committees of the Shandong Provincial Hospital, the Cheeloo College of Medicine and Shandong University, Jinan, Shandong, China. (NSFC: NO.2020‐913).

### 2.2 DMED model

DM was induced by a single intraperitoneal injection of 60 mg/kg STZ (Sigma‐Aldrich) in 30 mice. Ten mice were given vehicle only (0.1 mol/L citrate–phosphate buffer, pH 4.5) and used as a sham group. One week after this streptozotocin injection, blood glucose levels were determined using a blood glucose meter (Accu‐Chek, Roche). Animals with blood glucose levels consistently greater than 16.7 mmol/L were considered DM mice. Twenty diabetic mice were established successfully. Twelve weeks after DM induction, all surviving DM mice were assessed for erectile function with use of the apomorphine (APO) test, an established method for evaluating erectile function in mice.^15^ Mice received a subcutaneous injection of 100 μg/kg APO in the dorsal neck region and the number of penile erections over the following 30 min were recorded. At the end, twelve mice with the negative results of APO tests were confirmed to have DMED. The DMED mice were divided randomly into two treatment groups: the DMED control group (DMED group, *n *= 6) and the experimental group (DMED + NAC group, *n* = 6). The mice in DMED + NAC group receive NAC injected intraperitoneal ally (IP) for 4 consecutive weeks at a dose of 150 mg/kg. The body weights and blood glucose levels of all mice were recorded every 2 weeks. The antioxidant, NAC, was purchased from Beyotime Biotechnology and dissolved in normal saline at a concentration of 100 mg/ml. The dose and route of NAC administration were based upon previous results demonstrating effective anti‐oxidative effects with this protocol.^16^

### 2.3 Erectile function assessment

In addition to the APO test described above, erectile function was also assessed through measures of ratios of intracavernous pressure (ICP)/mean systemic arterial pressure (MAP) in response to electrical stimulation of the cavernous nerve (CN). ICP and MAP were measured as described previously.^17^ Briefly, mice were anaesthetized using a pentobarbital injection (35 mg/kg) and the left carotid artery was exposed, cannulated using PE‐50 tubing and a 25‐gauge needle was inserted into the left penile crura. The PE‐50 tubing and 25‐gauge needle were connected to a pressure transducer to measure MAP and ICP, respectively. The cavernous nerve was then electrically stimulated at 5 V and 15 Hz with a pulse width of 1.2 ms for 1 min at 3‐min intervals as described previously.^18^ The ratios of ICP to MAP were then used for calculations to assess erectile function.

### 2.4 Confocal immunofluorescence assay

We followed the methods of Li et al.^19^ Six mice were anaesthetized with pentobarbital sodium (Sigma‐Aldrich, P3761; 60 mg/kg body weight) and perfused with 4% paraformaldehyde (PFA). The penis was dissected, and samples were post‐fixed in PFA overnight at 4°C followed by a graded dehydration. The samples were then cut into serial coronal frozen slices (30 μm) with use of an oscillating blade microtome (Leica VT1200). Slices were incubated with primary polyclonal anti‐active caspase 3 (1:300, Abcam) and anti‐ionized calcium‐binding adaptor molecule‐1 (Iba‐1) (1:500, WAKO) followed by the Alexa Fluor488‐conjugated goat anti‐rabbit IgG or Alexa Fluor594‐conjugated goat anti‐mouse IgG secondary antibody (all 1:200, Sigma‐Aldrich). For analysis of ROS production, slices were stained with 10 μM dihydroethidium (DHE, Sigma) at 37°C for 30 min. Mitochondrial ROS levels were determined with use of 10 μM MitoSOX Red fluorescent dye applied for 15 min at room temperature in the dark. Hoechst 33258 (C0031), purchased from Solarbio, was applied for 5 min. Images were captured using a scanning laser confocal microscope (LSM780, Carl Zeiss). For fluorescent intensity quantification, 20× confocal images of fluorescent stained slides were analysed using Image‐Pro Plus. The integrated optical density (IOD) of IHC signals was divided by the area of DAPI signals to derive a fluorescent signal intensity. Fluorescent intensities were expressed as a per cent of the control group. A minimum of six to eight images were selected from each mouse for analysis.

### 2.5 Real‐time quantitative PCR analysis (qPCR)

Real‐time quantitative PCR analyses were used to detect mRNA expression levels. Total RNA was extracted using miRNA Isolation Kits (Beijing Tiangen Biochemical Technology Co., Ltd.). Real‐time quantitative PCR (RT‐qPCR) was performed on ABI7500 (Thermo Fisher Scientific) using special primers. The OD260/280 absorbance ratios were between 1.8 and 2.0 for all samples. The cDNA was synthesized by HiScript II Q Select RT SuperMix for qPCR (R233) (Vazyme); qPCR analysis was performed on StepOne Plus Real‐Time PCR system (Applied Biosystems). The PCR program included 95°C for 10 min, 40 cycles of 95°C for 10 s, 60°C for 20 s and 72°C for 34 s. The relative expressions of mRNA were calculated using the 2^−ΔΔCt^ method. β‐actin was used as an endogenous control to normalize each sample. PCR amplification efficiencies were established by means of calibration curve. The primers were shown as followed: IL‐10 (forward 5′ AGGCTACGGCGCTGTCATC3′; reverse 5′GGCATTCTTCACCTGCTCCA3′), IL‐1β (forward 5′TACATCAGCACCTCACAAGC3′; reverse 5′AGAAACAG TCCAGCCC ATACT3′), IL‐6 (forward 5′ATGCAAT AACCACCCCT 3′; reverse 5′AGTGTCCTAACGCTCATAC3′), TNF‐α (forward 5′GTG ACAAGCCTGTAGCCCA3′; reverse 5′ACTCGGCAAAGTCGAGATAG3′), bcl‐2 (TCGCAGAGATGTCCAGTCAG3′; reverse5′ATCTCCCTGTTGACGCTCTC3′), Bax (forward 5′TGAAGACAGGGGCCTTTTTG3′; reverse 5′AATTCGCCGGAGACACTC G3′), caspase 3 (forward 5′TGGTGATGAAGGGGTCATTTATG3′; reverse 5′TTCGGCT TTCCAGTCAGACTC3′), caspase 9(forward5′CTGTCTACGGCACA GATGGAT3′; reverse 5′GGGACTCGTCTTCAGGGGAA3′), caspase 12(forward 5′CAAAGGTTTGGCCAAGGACAT3′, reverse 5′GCCT TGTTCAGGATGAAACTCG3′), p53(forward 5′GCGTG TGGAGTAT TTGGA3′, reverse 5′GAGAGGAGCTGG TGTTGTT3′) and GAPDH (forward 5′AAATGGTGAAGGTCGG TGTG3′; reverse 5′AGGTCA ATGAAGGGGTCGTT).

### 2.6 Western blot analysis

Penile tissue was homogenized in RIPA lysis buffer. The tissue was dispersed using ultrasound, centrifuged and the supernatant collected. Protein concentrations were determined using the BCA assay kit (Beyotime). Equal amounts (30 mg of protein extract/lane) of protein from each sample were electrophoretically separated on 12% SDS‐PAGE gels and transferred to PVDF membranes for analysis. After blocking for 1.5 h using bovine serum albumin at room temperature, the membranes were incubated with antibodies overnight at 4°C. Primary antibodies included polyclonal rabbit anti‐CD45 (1:1000, Abcam), anti‐CD11b (1:1000, Abcam), anti‐Nrf2 (1:1000, Abcam), anti‐HO‐1 (1:1000, Abcam), anti‐NF‐κB(p65) (1:1000, Abcam), anti‐P38 (1:1000, Abcam), anti‐p‐P38 (1:1000, Abcam), anti‐eNOS (1:1000, Abcam), anti‐p‐eNOS (1:1000, Abcam) and anti‐β‐actin (1:8000, Santa Cruz Biotechnology). The secondary antibody was horseradish peroxidase‐conjugated to mouse anti‐rabbit/mouse IgG (1:5000, Santa Cruz Biotechnology). Immunoblots were developed by Enhanced Chemiluminescence (ECL). Protein band densities were quantified using Image J software (NIH, Scion Corpomiceion) and were normalized to β‐actin. Final data were expressed as a per cent of controls.

### 2.7 Oxidative stress measurements

Activity of antioxidant enzymes within penile tissue was determined with use of the superoxide dismutase (SOD) activity (No. A001‐3) and Catalase (CAT) assay (No. A007‐1) kits. Antioxidant activity in penile tissue was determined using the Total antioxidant capacity (T‐AOC) assay kit (No. A015‐3‐1). These measurements were performed using tissue homogenates with standard protocols. Briefly, the substmicee stock solution with buffer was diluted to a ratio of 1:200, and the enzyme stock solution with enzyme was diluted to a ratio of 1:10. These solutions were then thoroughly mixed to enable complete contact between samples and reagents, which were then incubated at 37°C for 20 min. Finally, the mixture was assessed using an enzyme‐labelled instrument at an absorbance of 450 nm. The contents of lactate dehydrogenase (LDH) (No. A020‐2‐2), malondialdehyde (MDA) (No. A003‐1) and nitric oxide (NO) (No. A013‐2) were determined using assay kits according to the manufacturers’ guidelines. The MDA analysis was performed using tissue homogenates with standard protocols. The mixed reagent was placed in a 95°C water bath for 40 min, cooled in tap water, centrifuged at 1000 *g* for 10 min and supernatants were transferred to cuvettes with a 1 cm light path to measure absorbance at 532 nm. For determinations of LDH and NO levels, nitmicee was converted to nitrite using aspergillus nitrite reductase and total nitrite was then measured with use of the Griess reagent. After a 10 min incubation period at room temperature, absorbance was determined at 543 nm using a spectrophotometer. Six mice per group were used for these experiments. All assay kits were purchased from Jiancheng Inc.

### 2.8 ELISA assay

The mouse TNF‐α ELISA Kit (CSB‐E04741m, CUSABIO), mouse IL‐1β (CSB‐E08054m, CUSABIO), mouse IL‐10 (CSB‐E04634m, CUSABIO) and mouse IL‐6 (CSB‐E04639m) were used to determine the concentrations of TNF‐α, IL‐1β, IL‐6 and IL‐10 in plasma, respectively, following the manufacturer's instructions.

### 2.9 Statistics

Data management and analysis were performed using the GraphPad Prism version 8.0 program (GraphPad Software), and results are presented as means ± standard deviations. Normality of the data distribution was assessed using the Kolmogorov–Smirnov test. One‐way anova was used for determinations of statistical significance, and Tukey test was used for post hoc analysis. *p* value < 0.05 was required for results to be considered as statistically significant.

## 3 RESULTS

### 3.1 NAC treatment improves erectile dysfunction in streptozotocin‐induced diabetes mellitus mice

**FIGURE 1:**
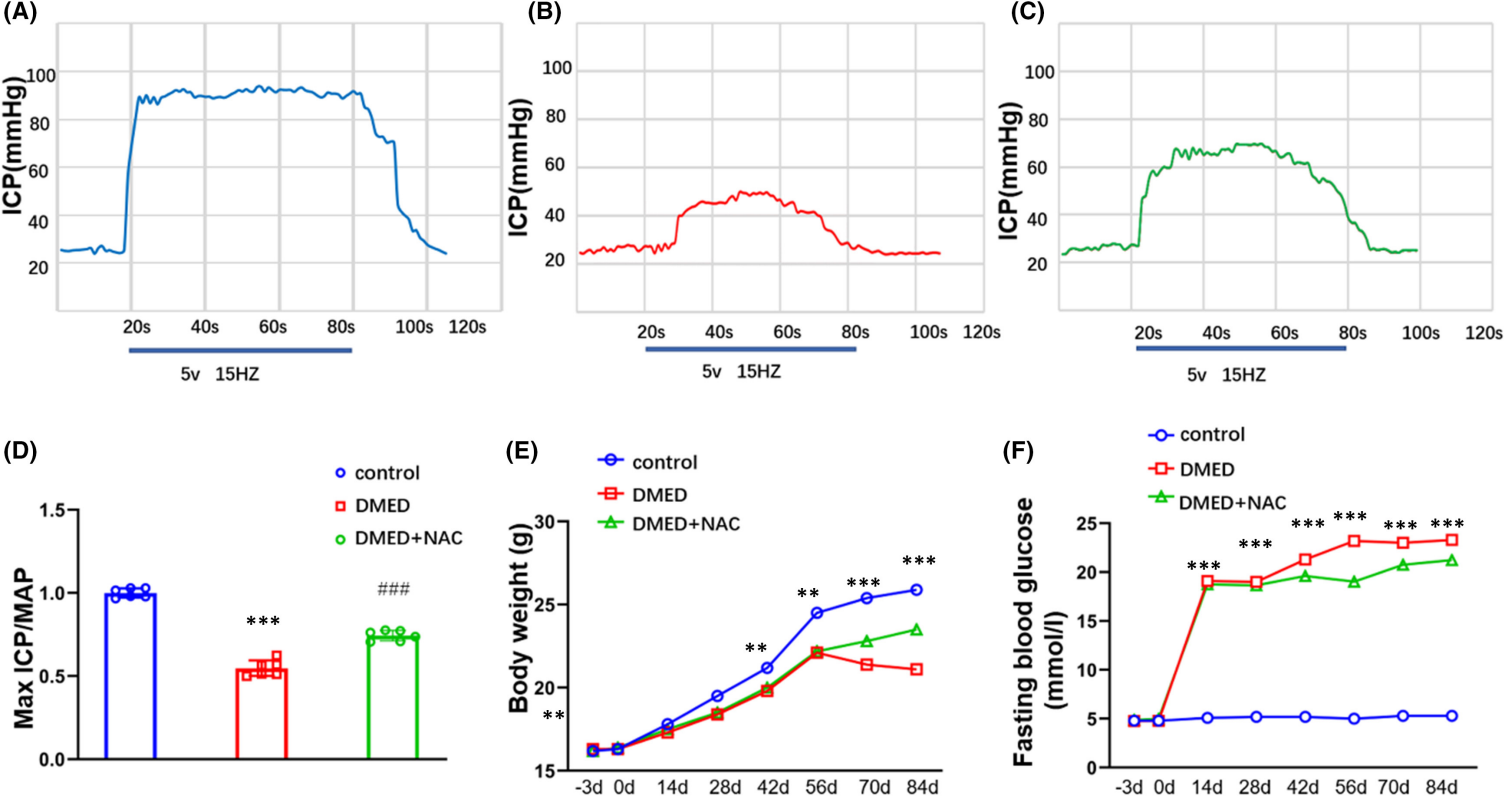
Comparisons of blood glucose levels and body weights between DMED and controls after STZ injection. (A) Representative traces of ICP in control mice (B) Representative traces of ICP in DMED mice. (C) Representative traces of ICP in DMED + NAC mice. (D) Maximal ICP/MAP ratios. (E) Body weights. (F) Blood glucose levels. The data are presented as means ± SDs. *N* = 6 per group. *p < 0.05 compared with control group. ^#^ *p *< 0.05 compared with DMED group. DMED, diabetes mellitus‐induced erectile dysfunction; ICP, intracavernous pressure; MAP, mean systemic arterial pressure; NAC, N‐acetylcysteine

Body weights and blood glucose levels were determined weekly after STZ injection within the DMED and control groups. Blood glucose levels were significantly increased (*p *< 0.05; Figure 1F), while body weights significantly decreased (*p *< 0.05; Figure 1E) in the DMED versus control group. In addition, maximal ICP and ICP/MAP ratios were significantly decreased in DMED versus control mice (*p *< 0.05 for both; Figure 1A–D).

### 3.2 NAC decreased oxidative stress in penile cavernous tissue of DMED mice

**FIGURE 2:**
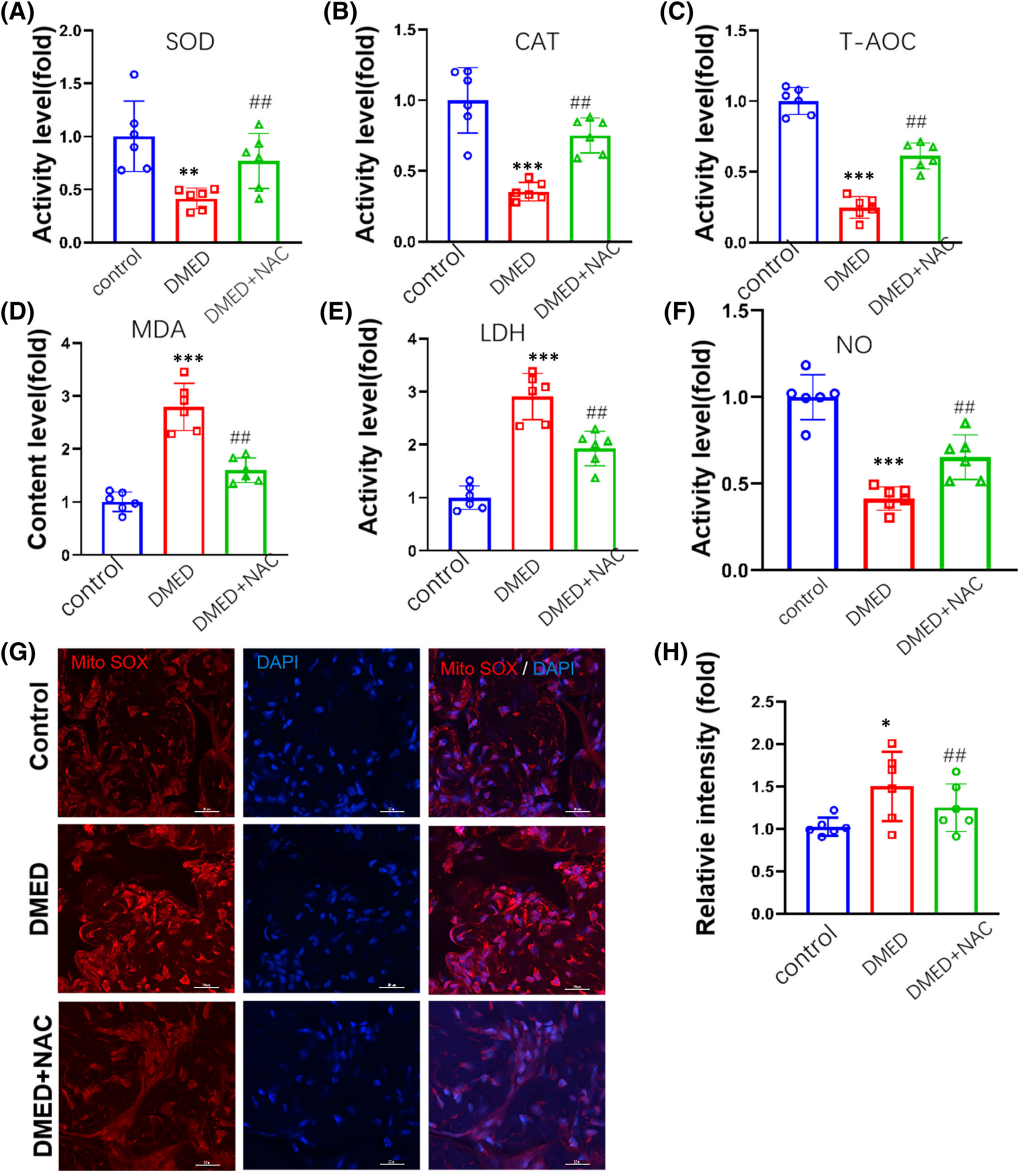
Comparisons of oxidative stress levels in the corpus cavernosum among control, DMED and DMED + NAC mice. (A) SOD activity. (B) CAT activity. (C) T‐AOC ability. (D) MDA contents. (E) LDH contents. (F) NO contents. (G) MitoSOX levels. (H) Quantitative analysis of relative intensities of Mito SOX‐positive cells. Nuclei (blue) are stained with DAPI. Scale bar is 20 μm. *N* = 6/group per group. CAT, catalase; LDH, lactate dehydrogenase; MDA, malondialdehyde; NO, nitric oxide; SOD, superoxide dismutase; T‐AOC, total antioxidant capacity. *p < 0.05, **p < 0.01 and ***p < 0.001, compared to the control group. ^#^ *p* < 0.05, ^##^ *p* < 0.01 and ^###^ *p* < 0.001, compared to the DMED group

To assess potential mechanism in penile tissue of DMED mice after NAC treatment, we first examined changes in oxidative stress levels. We found that activities of the antioxidant enzymes, SOD and CAT, were significantly decreased in DMED versus control mice. However, after NAC treatment, the activities of SOD and CAT were significantly increased (*p *< 0.05 for both; Figure 2A,B). In addition, T‐AOC was also decreased in DMED mice (*p* < 0.05; Figure 2C). Levels of the oxidative stress products, MDA, were significantly increased in DMED as compared with that of the nondiabetic control group. However, the level of MDA was decreased in DMAD + NAC group. (*p* < 0.05; Figure 2D). Moreover, the findings that levels of LDH were significantly increased in DMED mice indicate that more serious damage was present within penile tissue in this group as compared with that of the control group. However, LDH was significantly decreased after NAC treatment (*p* < 0.05; Figure 2E). Levels of the NO were significantly decreased in DMED as compared with that of the nondiabetic control group. However, the level of NO was increased in DMAD + NAC group (*p* < 0.05; Figure 2F).

This accumulation of oxidative stress products as observed in DMED mice can result in mitochondrial and DNA damage, thus leading to endothelial cell death in the penis. Notably, measures of MitoSOX, which provide an index of mitochondrial superoxide levels, were significantly increased in DMED versus control mice. However, the MitoSOX level was decreased after NAC treatment (*p* < 0.01; Figure 2G,H).

### 3.3 NAC decreased inflammatory responses in penile cavernous tissue of DMED mice

**FIGURE 3:**
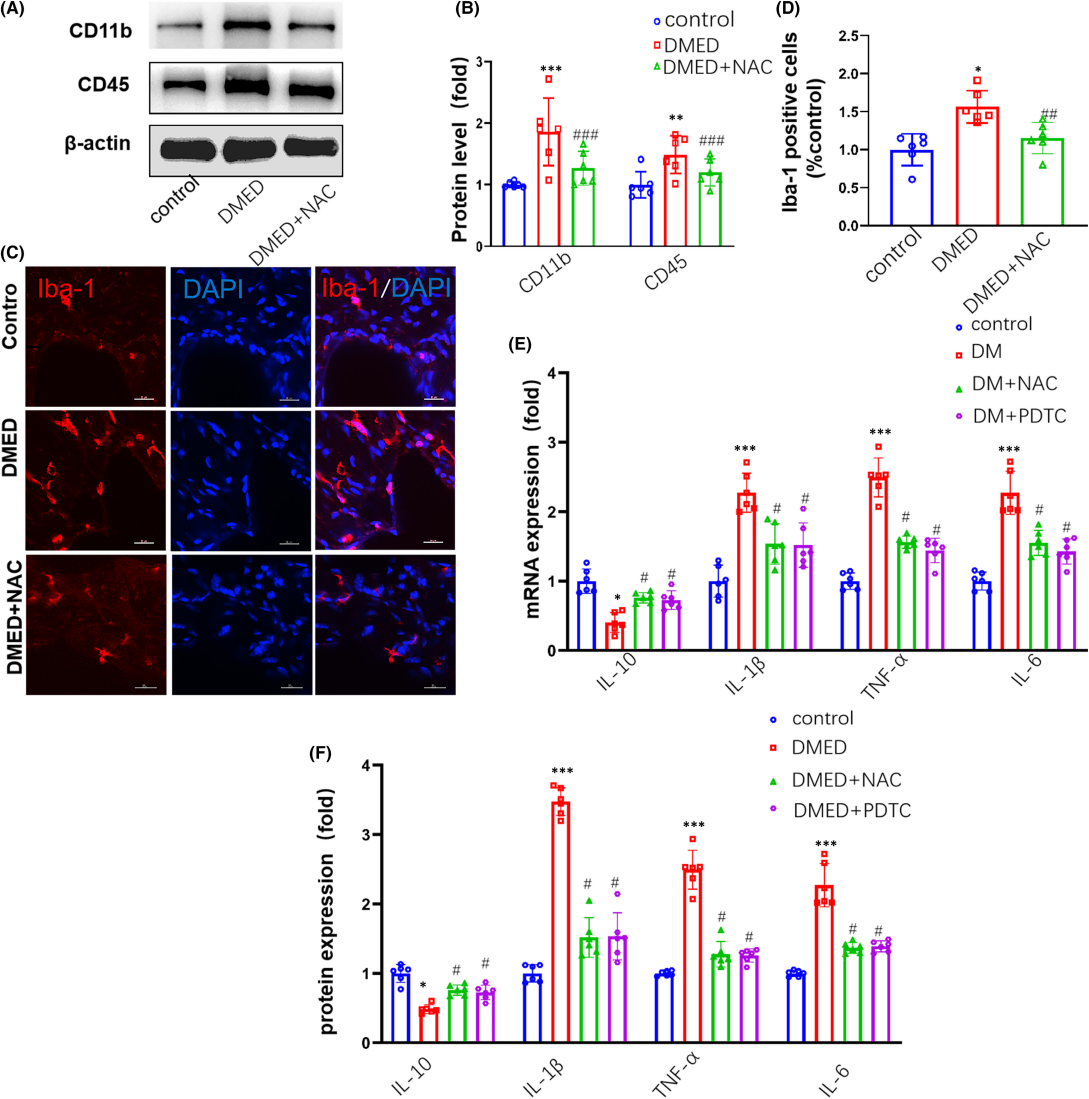
Comparisons of inflammatory cytokine expressions in the corpus cavernosum among control, DMED and DMED + NAC mice. (A) Representative Western blot for CD45 and CD11b. (B) Western blot analysis of CD45 and CD11b protein expressions. (C) Immunofluorescent signals of Iba‐1‐positive microglial cells. Scale bar is 20 μm. (D) Quantitative analysis of relative intensities of Iba‐1‐positive cells. (E) qPCR assay results for mRNA expression levels of IL‐10, IL‐1β, TNF‐α and IL‐6. (F) ELISA analysis results for protein levels of IL‐10, IL‐1β, TNF‐α and IL‐6. PDTC: pyrrolidine dithiocarbamate. *N* =6 per group. *p < 0.05, **p < 0.01 and ***p < 0.001, compared to the control group. ^#^ *p* < 0.05, ^##^ *p* < 0.01 and ^###^ *p* < 0.001, compared to the DMED group

Inflammation is considered a critical risk factor in the pathogenesis of DM. NAC could effectively reduce inflammation. Here, results from Western blot analysis revealed that an over‐expression in the proteins, CD11b and CD45, which represent important inflammation markers in diabetic, were observed in DMED mice. However, the protein levels of CD11b and CD45 were lower in DMED + NAC than in DMED group (*p* < 0.05; Figure 3A,B). Consistently, the number of Iba‐1‐positive cells in DMED mice was found to be significantly greater than the control groups. However, the number of Iba‐1‐positive cells in DMED + NAC mice was significantly lower than the DMED groups (*p* < 0.001, Figure 3C,D). In order to explore the mechanism of NAC reducing the inflammatory response, we added a group of DMED mice injected with PDTC (NF‐κB inhibitor) mice (DMED + PDTC group), we found that mRNA expressions and protein levels of several essential proinflammatory cytokines, such as IL‐1β, IL‐6 and TNF‐α, were all significantly increased in DMED as compared to that observed in control mice, while the anti‐inflammatory cytokine, IL‐10, was significantly decreased. However, the levels of IL‐1β, IL‐6 and TNF‐α were lower in DMED + NAC group and DMED + PDTC group than in DMED group (*p* < 0.05; Figure 3E).

### 3.4 NAC decreased apoptosis in penile cavernous tissue of DMED mice

**FIGURE 4:**
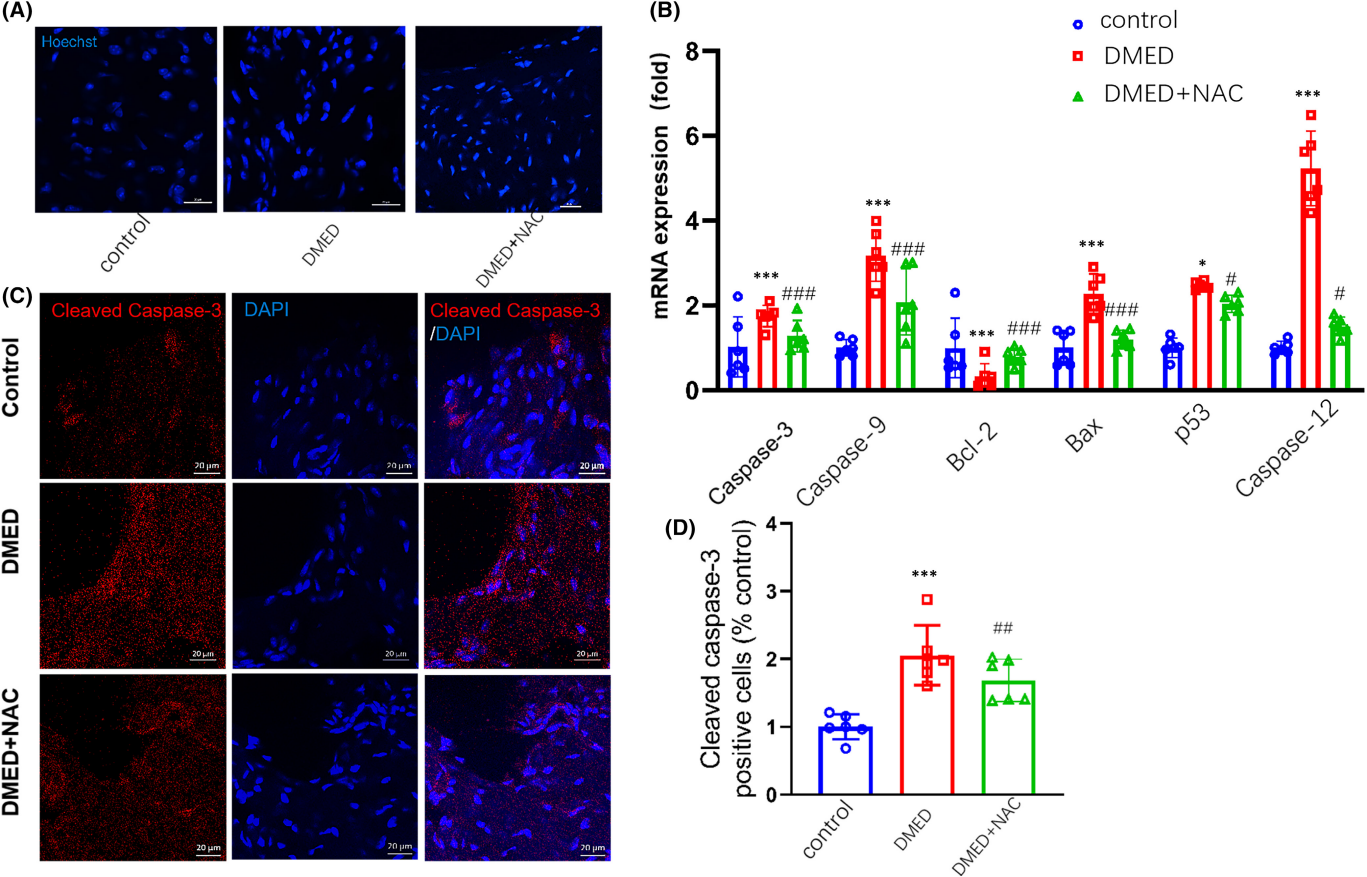
Comparisons of apoptosis in the corpus cavernosum among control, DMED and NAC‐treated mice. (A) Representative images of Hoechst‐33258 staining for observation of morphological changes in nuclei of DMED mice showing increases in apoptotic nuclei. Scale bar is 20 μm. (B) qPCR assay results for mRNA expression levels of Bcl2, Bax, caspase 3, p53, caspase 9 and caspase 12. (C) Immunofluorescent signals of cleaved caspase 3‐positive cells. Scale bar is 20 μm. (D) Quantitative analysis of relative intensities of cleaved caspase 3‐positive cells. *N* = 6 per group. ***p < 0.001, compared to the control group. ^##^ *p *< 0.01 and ^###^ *p* < 0.001, compared to the DMED group

Increased activation of oxidative stress and/or inflammation may result in cell death or senescence. Results from Hoechst staining revealed that DMED mice exhibited nuclear chromatin margination, aggregation and condensation, all changes which are typical of apoptotic nuclei (Figure 4A). We found that mRNA levels of the apoptosis‐related proteins Bax, caspase 3, p53, caspase 9 and caspase 12 were all significantly increased, while the apoptosis‐inhibiting protein, Bcl2, was significantly decreased in DMED mice as compared with controls. However, in DMED + NAC group, the mRNA levels of Bax, caspase 3, p53, caspase 9 and caspase 12 were lower than that in DMED group (*p* < 0.001; Figure 4B). In addition, the density of positive cleaved caspase 3 cells was significantly increased in DMED mice. However, the density of positive cleaved caspase 3 cells was significantly decreased in DMED + NAC mice (*p* < 0.01; Figure 4C,D).

### 3.5 NAC decreased oxidative stress and inflammation via Nrf2/HO‐1 and P38/NF‐κB(p65) pathways in DMED mice

**FIGURE 5:**
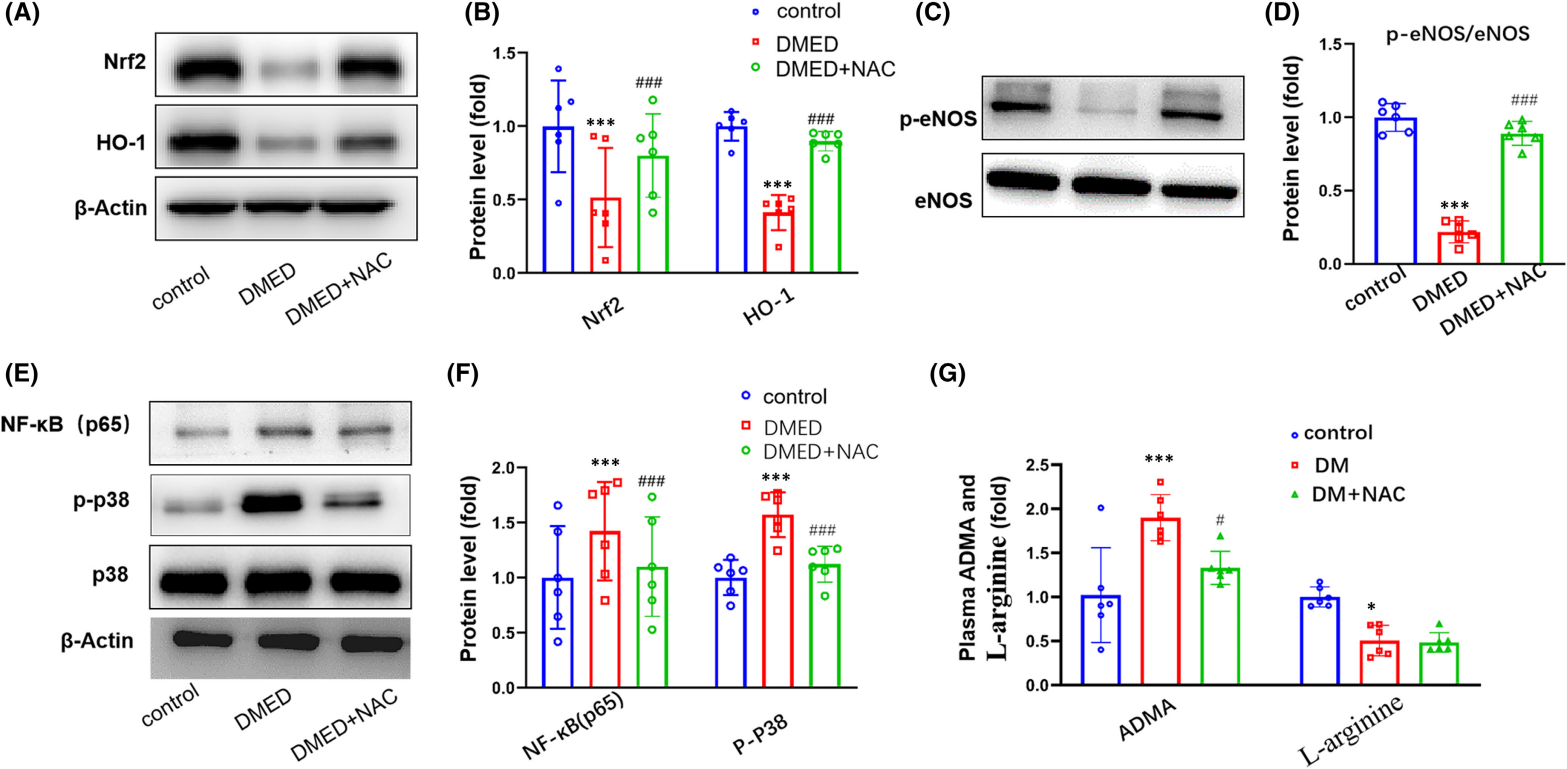
Comparisons of Nrf2, HO‐1, P38 and NF‐κB (p65) protein levels in the corpus cavernosum among control, DMED and NAC‐treated mice. (A) Representative Western blot of Nrf2 and HO‐1. (B) Western blot analysis of Nrf2 and HO‐1 protein expressions. (C) Representative Western blot of eNOS and p‐eNOS. (D) Western blot analysis of eNOS and p‐eNOS protein expressions. (E) Representative Western blot of NF‐κB(p65) and p‐P38. (F) Western blot analysis of NF‐κB(p65) and p‐P38 protein expressions. (G) Representative plasma ADMA and L‐Arginine levels. ADMA, asymmetric dimethylarginine. *N* = 6 per group. ***p < 0.001, compared to the control group. ^###^ *p *< 0.001, compared to the DMED group. * *p* < 0.05, compared to the control group. ^#^ *p *< 0.05, compared to the DMED group

NAC increased the ratio of p‐eNOS via inhibiting ADMA concentration. The Nrf2/HO‐1 pathway represents a key mechanism involved with inhibiting oxidative stress in human tissues. The phosphorylation of p38 can promote the expression of a number of inflammatory cytokines, while NF‐κB(p65) is a key factor regulating the expression of inflammatory factors. As shown in Figure 5A, there is a decrease in cavernous Nrf2 and HO‐1 protein levels within DMED mice, which indicates an increase of oxidative stress. However, in DMED + NAC group, the levels of Nrf2 and HO‐1 protein were higher than that in DMED group (*p* < 0.001; Figure 5A,B). Protein expressions of p‐eNOS were decreased in DMED as compared with the control group, However, in DMED + NAC group, the levels of p‐eNOS protein were higher than that in DMED group (*p *< 0.01; Figure 5C,D). Protein expressions of NF‐κB(p65) and p‐P38 were also increased in DMED as compared with the control group, indicating an increase in inflammatory responses were present in the penis tissue of the former (*p* < 0.001; Figure 5E,F). However, in DMED + NAC group, the levels of NF‐κB (p65) and p‐P38 protein were lower than that in DMED group. As shown in Figure 5G, the concentration of ADMA was higher and the concentration of L‐Arginine was lower in DMED as compared with the control group. However, in DMED + NAC group, the levels of ADMA were lower than that in DMED group (*p *< 0.01). There was no significant difference of L‐Arginine levels between DMED group and DMED + NAC group.

## 4 DISCUSSION

In the present study, we attempted to explore the therapeutic effect of NAC and the underlying molecular mechanisms in a STZ‐induced mouse model of diabetes mellitus with erectile dysfunction (DMED). The key finding of our study was that NAC significantly improved erectile function through inhibiting oxidative stress, inflammatory factors and apoptosis in the penile tissue. Our results also revealed that the oxidative stress and inflammatory factors in the penile tissue of these DMED mice were significantly elevated as compared with controls, an effect which resulted in increased apoptosis in the penile tissue, leading to abnormalities in erectile function. Taken together, these findings suggest that future developments for therapies directed towards treating DMED patients will require procedures which can mitigate inflammation‐oxidative stress responses within the microenvironment of the penis.

Results from previous studies have indicated that oxidative stress, as characterized by an overproduction of ROS, plays an important role in the pathogenesis of DMED.^20^, ^21^, ^22^ Increased ROS production, with its generation of the free radical superoxide anion (O_2_^−^), can react with NO to form the peroxynitrite anion (ONOO^−^), thereby diminishing NO bioavailability.^23^, ^24^ In addition, the ROS product, hydrogen peroxide, represents a superoxide capable of inducing endothelial cell injury by impairing intracellular constituents such as proteins, lipids and nucleic acids.^25^ NAC is an antioxidant and can inhibit the inflammation that occurs in many pathological conditions,^26^ but there are few studies on the effect of NAC on DMED. In the present study, we focused our attention on investigating changes within the cavernosal microenvironment of DMED mice after NAC treatment. With this model, we show that there are decreases in antioxidant enzymes, such as SOD and CAT, and increases in oxidative stress products, such as T‐AOC and MDA. Such results suggest that these changes in oxidative stress responses within the local microenvironment of the penis may be critical components contributing to ED. NAC could significantly decrease oxidative stress, which was similar to other studies about NAC.^27^ With excessive ROS production, mitochondrial oxidative stress is enhanced as revealed by elevated levels of MitoSOX. To further assess some of the potential underlying mechanisms involved, we focused on the Nrf2/HO‐1 signalling pathway, which is a classic oxidative stress pathway.^28^ Nrf2 is an important transcription factor against oxidative stress, such that increases in oxidative stress, enables Nrf2 binding to an antioxidant response element (ARE), such as HO‐1.^29^ As a result of this process, multiple antioxidant proteins are activated which can then protect cells against oxidative damage.^30^ Our results show that expressions of Nrf2 and HO‐1 in DMED mice were significantly increased after NAC treatment as compared with that in DMED mice, indicating that NAC dramatically inhibited oxidative stress damage in DMED mice via Nrf2/HO‐1 pathway, which was similar to previous studies.^31^ Increasing evidence suggests that ADMA is associated with diabetic cardiovascular complications. Endogenous ADMA accumulation was augmented resulting in decreased nitric oxide (NO) production and increased oxidative stress. In addition, DMED mice exhibit high levels of ADMA, a molecule with inhibitory action on nitric acid synthetase (NOS) and low levels of the endothelium‐derived relaxant factor NO. After NAC treatment, the level of ADMA decreased, and the levels of NO and eNOS increased in DMED mice. This is similar to the results of previous studies on NAC.^32^, ^33^

ROS can also induce activation of the NF‐κB protein, which then further promotes the triggering of inflammatory cytokines, such as TNF‐α and IL‐6.^34^ The homeostatic imbalance in vascular physiology would then result in endothelial cell damage and endothelial dysfunction.^35^ The increases in inflammatory factors, such as IL‐1β, IL‐6 TNF‐α and CD11b and CD45 proteins, as observed in these DMED mice, also suggests that enhanced inflammatory responses may contribute to this ED.^36^ NAC significantly reduced the expression of these inflammatory factors,^31^ effectively alleviated the inflammation of the penis and promoted the recovery of erectile function. Moreover, as reported in previous studies, a continuous presence of inflammatory responses and oxidative stress increases apoptosis, which is similar to the findings of our current results.^37^ While endothelial cells maintain vascular endothelial function by secreting a variety of cytokines,^38^ hyperglycaemia induces death or apoptosis in these endothelial cells, directly leading to vascular endothelial dysfunction.^39^ An accumulation of ROS also affects several intracellular pathways associated with vascular endothelial function, such as decreased eNOS expression or activity, resulting in poor endothelium‐dependent vasorelaxation and ED.^40^ Additional mechanisms may also represent significant components contributing to ED. For example, chronic inflammation and oxidative stress impair neurological systems, such as non‐adrenergic non‐cholinergic nerves (NANC), which can then decrease nNOS activity and corporal relaxation dysfunction.^13^ Moreover, the release of ROS induces oxidative stress leading to abnormal gene expression, faulty signal transduction and apoptosis of cells^41^; and hyperglycaemia also induces apoptosis by p53 and via activation of the cytochrome c‐activated caspase 3 pathway. Such processes are consistent with our current experimental results.^42^

There are some limitations of this study. It should be noted that DMED occurs in both type 1 as well as type 2 diabetes and the STZ‐induced diabetic model is a better reflection of type1 diabetes. Consider of the way to conduct T1DM model mice is more quickly and easier than T2DM model and the main pathogenesis of DMED is persistent hyperglycaemia, we select this model. In addition, the method of multiple low‐dose approach increased the accidental delivery into the bowel or sub‐dermal space may result in increased mortality or decreased diabetogenic effect. Taking this into account, we used a single high‐dose STZ injection to reduce the risk of complications from multiple injections. Moreover, in this report, we focused on the Nrf2 pathway of oxidative stress within this DMED model, as this may represent one of the more critical pathways involved. However, other oxidative stress‐related pathways may also be involved and will require investigation in future studies. Finally, DMED is a complex condition involving potential interactions among neuroregulatory and endocrine‐related factors. Although our current results have unravelled some of this complexity, our experiments failed to consider the impact of neuroregulatory factors, which could also represent an important influence in the complete characterization of ED.

## 5 CONCLUSION

This study demonstrated that NAC improved erectile function in DMED mice through inhibiting oxidative stress via Nrf2/HO‐1 pathway and reducing inflammation through MAPK p38/NF‐κB pathway in penile cavernous tissue. In addition, NAC increased NO and eNOS via decreasing ADMA concentration. NAC also protected the endothelial cell apoptosis. NAC might provide a new promising therapeutic strategy for individuals with DMED.

## AUTHOR CONTRIBUTION

**Zhen Ma:** Data curation (equal); Methodology (equal); Software (equal); Writing – review & editing (equal). **Wenzhen Wang:** Data curation (equal). **Chao Pan:** Formal analysis (equal). **Cuiqin Fan:** Investigation (equal); Methodology (equal); Supervision (equal). **Ye Li:** Software (equal); Supervision (equal). **Wenjing Wang:** Validation (equal); Visualization (equal). **Tian Lan:** Data curation (equal); Resources (equal). **Fangxin Gong:** Methodology (equal); Software (equal). **Changbo Zhao:** Supervision (equal); Validation (equal). **Zichao Zhao:** Software (equal). **Shuyan Yu:** Conceptualization (equal); Investigation (equal); Supervision (equal); Writing – original draft (equal). **Mingzhen Yuan:** Conceptualization (equal); Validation (equal); Visualization (equal); Writing – original draft (equal).

## CONFLICTS OF INTEREST

The authors declare no conflict of interest.

## Data Availability

The data that support the findings of this study are available from the corresponding author upon reasonable request.
